# Evaluation of uterine scar healing by transvaginal ultrasound in 607 nonpregnant women with a history of cesarean section

**DOI:** 10.1186/s12905-021-01337-x

**Published:** 2021-05-13

**Authors:** Xingchen Zhou, Tao Zhang, Huayuan Qiao, Yi Zhang, Xipeng Wang

**Affiliations:** 1 Department of Obstetrics and Gynecology, Xinhua Hospital, Affiliated to Shanghai Jiaotong University, No. 1665 Kong Jiang Road, Shanghai, 200092 China; 2 Department of Gynecology, Qingdao Municipal Hospital, Shandong, 266071 China; 3 Department of Ultrasound, Shanghai First Maternity and Infant Hospital, Affiliated To Tongji University, Shanghai, China

**Keywords:** Cesarean section, Cesarean scar defect (CSD), Thickness of residual myometrial (TRM), Transvaginal ultrasound (TVS), Predictive model

## Abstract

**Background:**

Caesarean scar defect (CSD) seriously affects female reproductive health. In this study, we aim to evaluate uterine scar healing by transvaginal ultrasound (TVS) in nonpregnant women with cesarean section (CS) history and to build a predictive model for cesarean scar defects is very necessary.

**Methods:**

A total of 607 nonpregnant women with previous CS who have transvaginal ultrasound measurements of the thickness of the lower uterine segment. The related clinical data were recorded and analyzed.

**Results:**

All patients were divided into two groups according to their clinical symptoms: Group A (N = 405) who had no cesarean scar symptoms, and Group B (N = 141) who had cesarean scar symptoms. The difference in frequency of CS, uterine position, detection rate of CSD and the residual muscular layer (TRM) of the CSD were statistically significant between groups; the TRM measurements of the two groups were (mm) 5.39 ± 3.34 versus 3.22 ± 2.33, *P* < 0.05. All patients were divided into two groups according to whether they had CSDs: Group C (N = 337) who had no CSDs, Group D (N = 209) who had CSDs on ultrasound examination. The differences in frequency of CS, uterine position, TRM between groups were statistically significant (*P* < 0.05). In the model predicting CSDs by TRM with TVS, the area under the ROC curve was 0.771, the cut-off value was 4.15 mm. The sensitivity and specificity were 87.8% and 71.3%, respectively.

**Conclusions:**

Patients with no clinical symptoms had a mean TRM on transvaginal ultrasonography of 5.39 ± 3.34 mm, which could be used as a good reference to predict the recovery of patients with CSDs after repair surgery.

## Background

With the increasing cesarean section (CS) rate, a growing number of studies suggest that the occurrences of long-term consequences of CS are related to the incomplete healing of the CS scar in the uterus, which leads to the development of cesarean scar defects (CSDs) [1, 2]. In routine ultrasound examinations of the uteri of nonpregnant women with a history of at least one CS, the prevalence of CS scar defects ranges from 24 to 70% [3]. Some patients with CSDs are asymptomatic, but several investigators have reported an association between these defects and abnormal bleeding and postmenstrual spotting [4, 5]; CSDs can also cause chronic pelvic pain and even infertility [6]. Moreover, the development of CSDs seems to be on the rise, and CSDs can occur on a spectrum of disorders starting with cesarean scar (CS) ectopic pregnancy [7], to increased incidence of placenta previa and uterine rupture associated with major maternal morbidity, and even mortality [8]; this is a very important medical problem that affects a large population of women. Therefore, the detection and diagnosis of uterine incision diverticulum is very important, especially in asymptomatic patients.

In particular, the thickness of the residual muscular layer (TRM) of the CSD directly affects the severity of the clinical symptoms and the risk of maternal complications, such as uterine rupture in subsequent pregnancy [8]. Therefore, it is very important to evaluate uterine scar healing in nonpregnant women with previous cesarean section. Transvaginal ultrasound is the most convenient and noninvasive means of examination. However, most of the studies about uterine incision healing included measurements of the lower uterine segment (LUS) thickness in pregnant women with a previous caesarean delivery [9, 10]. There is a lack of transvaginal ultrasound evaluations and multisampling statistical analyses for LUS measurements in nonpregnant women. There is no clinical reference standard to evaluate the efficacy of surgical repair in CSD patients. Therefore, this study investigated the ability of transvaginal ultrasound to detect cesarean scars and the prevalence of scar defects in nonpregnant subjects. By measuring the thickness of the scar in the lower segment of the uterus, diameters of the CSDs, uterine position and other imaging data, the range of TRM values in nonpregnant women with a CS history could be determined. The purpose of this study was to determine the value of LUS thickness that predicts good healing of the uterine incision after cesarean section and to reveal the clinical indicators that predict CSD and could be used clinically in patient management.

## Methods

### Patients selection

In this prospective study, 607 patients who presented at the Shanghai First Maternity and Infant Hospital, Tongji University from October 2016 to October 2018 were enrolled. Inclusion criteria: All patients had undergone at least one C-section and were currently not pregnant with or without a prolonged menstruation period. Exclusion criteria: (1) Patients had a history of other uterine surgery that could have changed their cavity anatomy. (2) The patient had a congenital uterine abnormality, such as a biangular uterus. (3) The patient had undergone classical cesarean section. All subjects underwent transvaginal ultrasonography (TVU) to assess uterine scar healing. This study was approved by the Ethics Committee of Shanghai First Maternity and Infant Hospital, affiliated with Tongji University (KS1512), and was conducted in accordance with the Declaration of Helsinki. All patients enrolled in the study signed informed consent forms.

### Methods

**Fig. 1:**
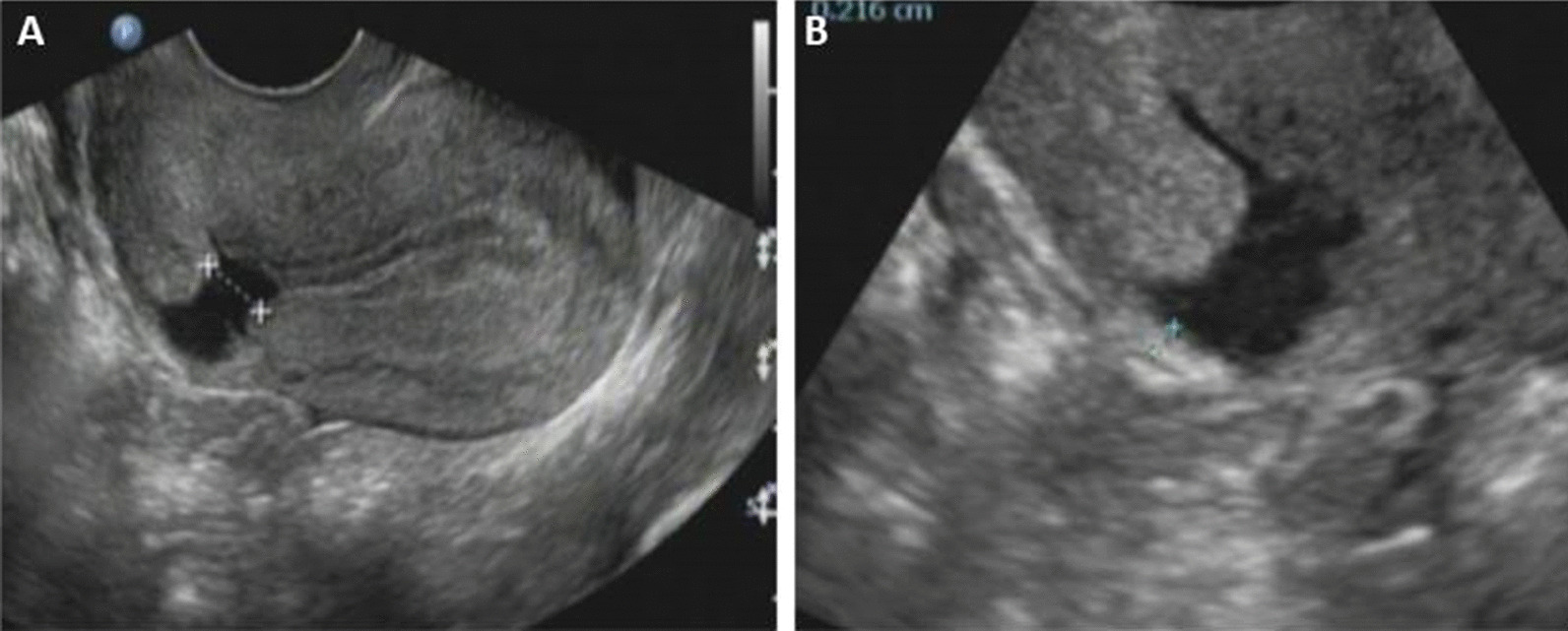
**a** CSD under transvaginal ultrasound; **b** TRM under transvaginal ultrasound

Each subject underwent a transvaginal ultrasound of the cervix, uterus, and adnexa, which was performed by one experienced sonographer blinded to the woman’s history. Ultrasound examinations were performed using a color ultrasonic diagnostic apparatus, Philips iU22, Philips HD15, GE E8. Both ultrasound devices were equipped with a 4–9 MHz transvaginal probe. Cesarean scar defects were recorded as either present or absent. If a CSD was present, the defect was measured in two dimensions in the sagittal plane (anterior–posterior and cephalad–caudal) and transversely in the coronal plane. Three different values of length, width, and depth as well as TRM were taken, and the mean value of these parameters was considered the actual data. The frequency of scar identification, as well as the presence of fluid within the scar (“scar defect”), was recorded and later compared with the self-reported obstetric history. Photo documentation was recorded with the presence or absence of a cesarean scar defect and the presence or absence of fluid within the scar (Fig. 1). Additional images of the endometrial stripe thickness, presence or absence of polyps or myomas, and ovarian volumes were also recorded. Each subject was assigned a study number at enrollment. Data obtained from the questionnaires (including age, contact information, number of cesarean sections, time of last cesarean section, menstruation and so on) and from the ultrasound were entered separately.

### Statistical analysis

Measurement data, such as length, width and depth as well as the TRM of the bottom of the CSD were expressed as the mean ± SD. Continuous variables were compared by paired t-tests. The results of them were used to trace out receiver operating characteristic (ROC) curves to predict CSDs. With the use of these curves, the threshold values of each variable were set. Variables that achieved statistical significance in the univariate analysis were subsequently included in the multivariate analysis. A *P* < 0.05 (two-tailed) was considered statistically significant. SPSS version 24.0 was used for all statistical calculations.

## Results

### Patient characteristics

A total of 607 consenting women with a history of at least one cesarean section were enrolled in the study over the period from October 2016 to October 2018; 17 subjects who did not undergo transvaginal ultrasound and 43 patients who underwent transvaginal ultrasound but did not have TRM measurements were excluded. A total of 546 women completed the study. All patients were divided into two groups according to their clinical symptoms: Group A (N = 405) included women who had no cesarean scar symptoms, and Group B (N = 141) included women who had cesarean scar symptoms, such as postmenstrual spotting, prolonged menstruation, and continuous brown discharge. The women were also divided into two groups according to their CSD status: Group C (N = 337) included women who had no CSDs on the ultrasound examination, and Group D (N = 209) included women who had CSDs on the ultrasound examination. The baseline characteristics of the 546 patients are presented in Table 1.

**Table 1:** Patient characteristics

Group	noCSD	CSD	Total
asymptomatic	310	95	405
symptomatic	27	114	141
Total	337	209	546

### Clinical characteristics of the patients in Group A and Group B

Group A (N = 405) included women who had no clinical symptoms. Group B (N = 141) included women who had cesarean scar symptoms, such as postmenstrual spotting, prolonged menstruation, and continuous brown discharge. The clinical data and uterine incision healing data were compared between the two groups. The results showed that the average age of the two groups of patients was 35.09 ± 5.32 versus 34.00 ± 4.83 years old, and the median age of the two groups was 34 years old. The number of women who only had one previous CS in Group A was 356 (87.9%) versus the 102 (72.3%) in Group B. The uterine positions of two groups of patients on ultrasound examination were compared, anterior position: 242 (59.8%) vs. 65 (46.1%); meso-position: 20 (4.9%) vs. 6 (4.3%); and retroposition: 143 (35.3%) vs. 70 (49.6%), *P* < 0.05. The detection rates of CSD in the two groups were dramatically different, 23.5% vs. 80.9%, *P* < 0.05. The length, depth, width and TRM of the CSD were all significantly different between the two groups (Table 2).

**Table 2:** Characteristics of clinical data between symptomatic group (Group A) and asymptomatic group (Group B)

	Group A (N = 405)	Group B (N = 141)	*P*
Age (y)	35.09 ± 5.32	34.00 ± 4.83	*P* = 0.148
Number of C-section deliveries			
One	356 (87.9%)	102 (72.3%)	*P* < 0.05
Two	46 (11.4%)	36 (25.5%)	
More than two	3 (0.7%)	3 (2.1%)	
Uterus position			
Anteflexion	242 (59.8%)	65 (46.1%)	*P* < 0.05
Meso-position	20 (4.9%)	6 (4.3%)	
Retroflexion	143 (35.3%)	70 (49.6%)	
Hysteromyoma	71 (17.5%)	15 (10.6%)	*P* = 0.053
CSD (N)	95 (23.5%)	114 (80.9%)	*P* < 0.05
CSD parameters			
Length (mm)	5.09 ± 2.31	5.96 ± 2.64	*P* < 0.05
Depth (mm)	6.62 ± 3.05	8.39 ± 3.72	*P* < 0.05
Width (mm)	8.89 ± 4.15	11.44 ± 4.98	*P* < 0.05
D/W	1.85 ± 1.55	3.84 ± 3.08	*P* < 0.05
TRM (mm)	5.39 ± 3.34	3.22 ± 2.33	*P* < 0.05

### Clinical characteristics of the patients in Group C and Group D

Group C (N = 337) included women who had no CSDs on transvaginal ultrasound, Group D (N = 209) included women who had detected CSDs on transvaginal ultrasound. The average age of two groups patients was 35.04 ± 5.41 (Group C) versus 34.44 ± 4.88 (Group D) years old, and the median age of two groups was 34 years old. The number of patients who only had one previous CS in Group C was 297 (88.1%) versus the 161 (77%) in Group D, *P* < 0.05. The uterine position of two groups on transvaginal ultrasound were compared, anterior position: 215 (63.8%) versus 92 (44%), meso position: 11 (3.3%) versus 15 (7.2%), and retroposition: 111 (32.9%) versus 108%, *P* < 0.05. The mean TRM measurements in the two groups were 6.54 ± 2.13 versus 4.21 ± 3.03, *P* < 0.05. The TRM in the non-CSD group was significantly thicker than that in the CSD group (Table 3).

**Table 3:** Characteristics of clinical data in non-CSD group and CSD group

	Group C (N = 337)	Group D (N = 209)	*P*
Age (y)	35.04 ± 5.41	34.44 ± 4.88	*P* = 0.355
Symptomatic	27 (8.0%)	114 (54.5%)	*P* < 0.05
Number of C-section deliveries			
One	297 (88.1%)	161 (77%)	
Two	38 (11.3%)	44 (21.1%)	*P* < 0.05
More than twice	2 (0.6%)	4 (1.9%)	
Uterus position			
Anteflexion	215 (63.8%)	92 (44%)	*P* < 0.05
Meso-position	11 (3.3%)	15 (7.2%)	
Retroflexion	111 (32.9%)	102 (48.8%)	
Hysteromyoma	61 (18.1%)	25 (12%)	*P* = 0.056
TRM (mm)	6.54 ± 2.13	4.21 ± 3.03	*P* < 0.05

### Receiver operating characteristic curves and logistic analysis

**Fig. 2:**
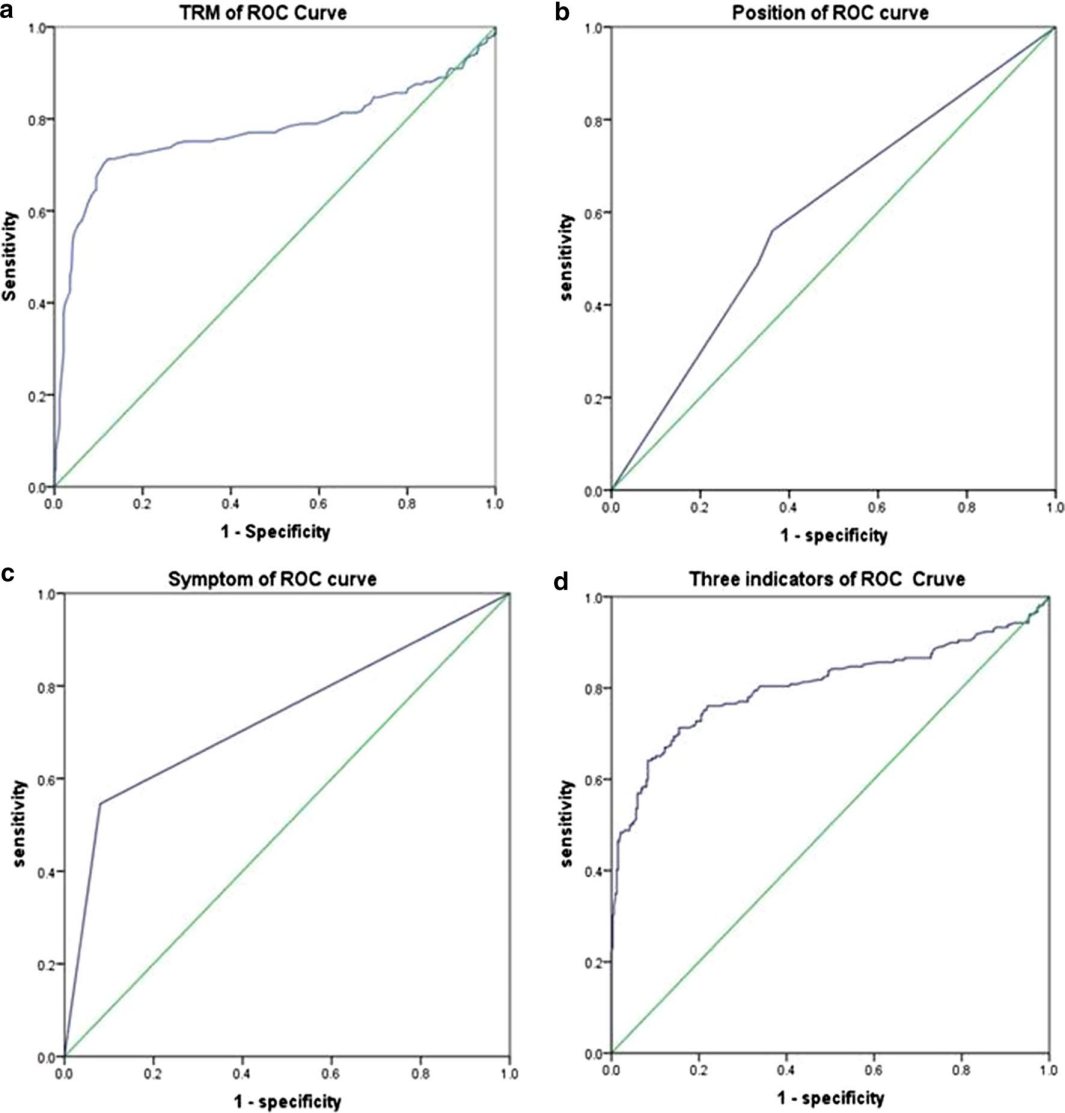
Receiver operating characteristic curves, **a** ROC curve of TRM thickness; **b** ROC curve of uterine position; **c** ROC curve of symptoms; **d** ROC curve of all three indicators

The clinical symptoms, uterine position, and TRM in the women with CSDs were significantly different from those in the women without CSDs. The results of logistic multivariate regression analysis are shown in Table 4. The ROC curves of the CSD and non-CSD groups were drawn with these three values (Fig. 2). The curves obtained from the three values together to predict CSD indicated a cutoff value of 0.346 with a sensitivity of 84.6% and a specificity of 55.9% (95% CI 0.76–0.85). In terms of individual indicators to predict CSDs, the TRM with TVS had a high predictive value. The ROC curve indicated that at the cutoff value of 4.15 mm, the TRM variable had a sensitivity of 87.8% and a specificity of 71.3% for predicting CSDs (95% CI 0.723–0.819), and the area under the ROC curve was 0.779 (Table 5).

**Table 4:** Logistic regression analysis results

	B	SE	Wald	df	*P*	Exp (B)	95% CI
Symptom	2.95	0.26	70.31	1	0.00	8.98	5.38–14.99
Uterus position	0.29	0.11	6.68	1	0.10	1.33	1.07–1.66
TRM	0.25	0.04	31.05	1	0.00	0.78	0.72–0.85

**Table 5:** Receiver operating characteristic curves

Indicator	Cut-off	AUC	*P*	Sensitivity	Specificity	Youden’s index	95% CI
Symptom	0.522	0.733	*P* < 0.05	0.545	0.92	0.465	0.686–0.779
Number of C-section deliveries	0.444	0.556	*P* < 0.05	0.23	0.881	0.111	0.506–0.606
Uterus position	0.389	0.603	*P* < 0.05	0.56	0.638	0.198	0.554–0.652
TRM	4.15	0.771	*P* < 0.05	0.878	0.713	0.591	0.723–0.819
Three indicators	0.346	0.805	*P* < 0.05	0.713	0.846	0.559	0.761–0.848

## Discussion

In China, the proportion of Caesarean sections (CS) performed In 2010 was 35–58%, which has also aroused widespread concern about CSDs [11]. With the increasing CS rate and the implemented two-child policy, the complications of CSDs, such as secondary infertility, prolonged menstrual bleeding, even uterine rupture during a subsequent pregnancy, have emerged as important clinical problems, which serious impact on women's reproductive health [12]. Therefore, it is necessary to evaluate cesarean section scar before the next pregnancy.

CSDs can be detected by transvaginal ultrasound (TVS) [2, 13], sonohysterography (SHG), hysterography, hysteroscopy (HSC), and magnetic resonance imaging (MRI) [14–17]. Among these methods, TVS is a simple,noninvasive and low-cost examination that should be considered as the first choice for screening CSDs [18], nevertheless, unskilled gynecologists or the use of a low-resolution ultrasound machine can miss defects during routine ultrasound scans, especially if the operator does not suspect a CSD and there does not look for a defect.

Singhl et al. [19] evaluated scar thickness in pregnant patients with previous caesarean section by TVS and magnetic resonance imaging (MRI) to determine the precision of radiologically measured scar thickness with the actual measured scar thickness. These measurements were correlated with each other and with the scar thickness measured during elective repeat caesarean section using a caliper. The study showed that the thickness measured with TVS had a better correlation coefficient with the actual scar thickness than the thickness measured with MRI (R = 0.72 vs. R = 0.59). Marasinghe’s research came to a similar conclusions [20]. These authors all relieved that TVS could be considered the preferred modality for antenatal scar thickness measurements.

Therefore, our study established a CSD risk assessment model by applying TVS to evaluate the uterine scar healing of 607 women with a history of cesarean section. The results showed that the TRM measured with TVS effectively predicted CSDs when TRM was less than 4.15 mm, and uterine incision diverticulum was more easily detected below this thickness threshold. In other words, if the detected TRM was less than 4.15 mm by ultrasonography, but a CSD was not found, it was suggested that the scar condition should be re-evaluated by other imaging examinations. This method could avoid missed diagnoses of poor uterine scar healing. But when the TRM was more than 4.15 mm, less than 5.39 mm, CSD can not be detect by TVS, there may be a small diverticulum and ultrasonic sensitivity cannot be detected, so we think this kind of diverticulum only cause menstrual blood accumulation but does not affect the thickness of the next pregnancy,meanwhile, when the thickness is greater than 5.39 mm, healing can be thought of a good, close to the normal muscle layer thickness. This theory still needs to be further verified by expanding the sample size in the future research.

A study by Hayakawa et al., in turn, the study involved 137 women demonstrated that double-layer interrupted sutures reduced the probability of poor myometrium healing after CS 30–38 days after surgery [21]. Another randomized study that enrolled 78 women with TRM evaluated by TVS after surgery found that suturing all the myometrial layers, including the endometrium, reduced the risk for poor healing and incomplete regeneration [22, 23]. A retrospective study by Sevket et al., which enrolled the longest follow-up period of 6 months after surgery, showed that the use of a double layer locked suture promoted complete healing [24]. Finally, a commentary assay on the evaluation of scar healing after CS points out that several research’s have also used 6–9 months after surgery to evaluate scar healing, indicating that 6 months after surgery represents a relatively stable state of scar healing [25]. This finding is in keeping with the follow-up results of our previous study about the transvaginal repair of CSDs, which showed that the wound healing was stable in 6 months after surgery [26]. In this study, women were followed up for more than 6 months after cesarean section.

Now, the clinical guidelines about the treatment of CSDs was remain unclear. Several successful surgical treatments for CSDs have been reported in recent years, including hysteroscopic resection, laparoscopic surgery, laparoscopic and hysteroscopic repair, and vaginal repair. In our previous studies, at 6 months after surgery, 80.3% of patients (94 of 117) reached ≤ 10 days of menstruation, 48 patients (63.2%) had no CSDs, and 11 patients (14.5%) had a > 70% reduction in CSD volume; additionally, CSDs still existed in more than 40% of patients after vaginal repair [27]. As long as the TRM increased and their menstrual symptoms improved, the repair surgery could still be considered effective in increasing the safety of the second pregnancy. However, no clinical guidelines have been issued for the management of CSDs with intermenstrual bleeding and/or thickness of the remaining muscular layer (TRM) or for the residual muscle thickness that is considered the ideal result of a repair. Therefore, we need to evaluate uterine scar healing in women after cesarean section to obtain the average level of scar recovery.

## Conclusions

This study showed that for patients with no clinical symptoms, the mean thickness of the TRM with transvaginal ultrasonography is 5.39 ± 3.34 mm; for the patients who have clinical symptoms, the mean thickness of the TRM with transvaginal ultrasonography is 3.22 ± 2.33 mm. We believe that a residual muscle thickness of 5.39 ± 3.34 mm could be used as a good reference to predict the recovery of patients with CSDs after repair surgery.

## Data Availability

The datasets generated and/or analyzed during the current study are not publicly available due to potential for individual and organizational privacy to be compromised. Reasonable requests for parts of the data will be considered by the corresponding author. The Shanghai First Maternity and Infant Hospital, Tongji University granted these permissions.

## Abbreviations

CS: Caesarean section
CSD: Caesarean scar diverticula (defect)
TRM: Thickness of the remaining muscular layer
TVU: Transvaginal ultrasound
GDM: Gestational diabetes mellitus
LUS: Lower uterine segment
ROC: Receiver operating characteristic
MRI: Magnetic resonance imaging
